# High prevalence of low bone mass and associated factors in Korean HIV-positive male patients undergoing antiretroviral therapy

**DOI:** 10.7448/IAS.17.1.18773

**Published:** 2014-01-09

**Authors:** Pyoeng Gyun Choe, Hyung Jin Choi, Nak-Hyun Kim, Wan Beom Park, Kyoung-Ho Song, Ji Hwan Bang, Eu Suk Kim, Sang Won Park, Hong Bin Kim, Myoung-don Oh, Nam Joong Kim

**Affiliations:** Department of Internal Medicine, Seoul National University College of Medicine, Seoul, Republic of Korea

**Keywords:** HIV, AIDS, osteopenia, osteoporosis

## Abstract

**Introduction:**

Low bone mass is prevalent in HIV-positive patients. However, compared to Western countries, less is known about HIV-associated osteopenia in Asian populations.

**Methods:**

We performed a cross-sectional survey in Seoul National University Hospital from December 2011 to May 2012. We measured bone mineral density using central dual energy X-ray absorptiometry, with consent, in male HIV-positive patients, aged 40 years and older. Diagnosis of low bone mass was made using International Society for Clinical Densitometry Z-score criteria in the 40–49 years age group and World Health Organization T-score criteria in the >50-year age group. The data were compared with those of a community-based cohort in Korea.

**Results:**

Eighty-four HIV-positive male patients were included in this study. Median age was 49 (interquartile range [IQR], 45–56) years, and median body mass index (BMI) was 22.6 (IQR, 20.9–24.4). Viral suppression was achieved in 75 (89.3%) patients and median duration of antiretroviral therapy was 71 (IQR, 36–120) months. The overall prevalence of low bone mass was 16.7% in the 40–49 years age group and 54.8% in the>50 years age group. Our cohort had significantly lower bone mass at the femur neck and total hip than HIV-negative Koreans in the 40–49 years age group. Low bone mass was significantly associated with low BMI, and a high level of serum carboxy-terminal collagen crosslinks, but was not associated with antiretroviral regimen or duration of antiretroviral therapy.

**Conclusions:**

Low bone mass is prevalent in Korean HIV-positive males undergoing antiretroviral therapy, and may be associated with increased bone resorption.

## Introduction

The advent of combination antiretroviral therapy (CART) has improved the prognosis of patients infected with HIV [1]. Long-term CART is associated with several metabolic complications including lipodystrophy, insulin resistance, diabetes and dyslipidemia [2]. It is also well known that low bone mass is prevalent in HIV-positive patients [3]. In one meta-analysis, osteoporosis was three times more prevalent among HIV-positive patients than among HIV-negative controls, and was especially common among those receiving antiretroviral therapy [4].

The prevalence of low bone mass may vary in different ethnic groups, and less is known about the characteristics of low bone mass in Asian HIV-positive patients than in Western patients. Considering that there is a marked predominance of men in the clinic where this study was conducted, and that gender is an important risk factor for low bone mass, we chose to include only male patients. The present study was undertaken to investigate the prevalence of, and risk factors for, low bone mass in Korean HIV-positive males undergoing antiretroviral therapy.

## Methods

### Study population

This cross-sectional study included HIV-positive male patients over 40 years old who underwent CART for at least three months at Seoul National University Hospital. A board-certified infectious disease specialist took a complete history from all participants. Demographic data (sex, ethnicity and date of birth), HIV exposure category (men who have sex with men [MSM], heterosexual), life style (smoking, alcohol consumption and physical activity), nadir CD4 cell count, current CD4 cell count and history of CART were recorded. Serum level of 25(OH) Vitamin D_3_, parathyroid hormone, carboxy-terminal collagen crosslinks (CTX), bone-specific alkaline phosphatase (ALP), total testosterone and free testosterone were measured for each participant. Serum CTX level was measured by electrochemiluminescence immunoassay using the Elecsys^®^ ß-Crosslaps serum assay kit (Roche Diagnostics). The study protocol was in accordance with institutional guidelines and approved by an institutional review board. Informed consent was obtained from the study participants.

### Measurements of anthropometric parameters and BMD

Height and body weight were measured by standard methods in light clothes. Body mass index (BMI) was calculated as weight divided by height squared (kg/m^2^). Bone mineral density (BMD) (g/cm^2^) measurements at central skeletal sites (lumbar spine, femoral neck and total hip) were obtained using dual energy X-ray absorptiometry (Lunar Prodigy, GE Medical System). Diagnosis of low bone mass was made using the International Society for Clinical Densitometry (ISCD) Z-score criteria (low BMD for chronological age, Z-score ≤ − 2.0) and the World Health Organization (WHO) T-score criteria (Osteopenia, −2.5 < T-score < − 1.0; Osteoporosis, T-score ≤ − 2.5) [5]. For comparison with the general population, the BMD of subjects in a representative Korean community-based cohort was used [6]. Both the HIV-positive study group and the HIV-negative general population group used the same Lunar Prodigy machine with the same software (Encore, GE) and the same manufacturer-provided Korean reference.

### Statistics

BMD differences were analyzed using Student's *t*-test. Risk factors for low bone mass were analyzed using a linear regression model with BMD as the dependent variable. Additional analysis was performed using binary logistic regression model with any BMD T-score < − 1.0 as the dependent variable and odds ratios per one standard deviation increment were shown for continuous variables. All significance tests were two-sided, and data analyses were performed with SPSS software (version 19.0; SPSS Inc., Chicago, IL, USA).

## Results

A total of 84 HIV-positive patients were included in this study. All were male, and 39 (46.4%) of them had the risk factors of homosexual behaviour. All were Korean, and median age was 49 years (IQR 45–56) (Table 1). Median CD4 cell count was 531 cells/µL (IQR, 345–762), and 75 (89.3%) patients had an HIV RNA level < 40 copies/mL. Median duration of antiretroviral therapy was 71 months (IQR, 36–120). Fifty-five (65.5%) of the subjects had vitamin D deficiency.

**Table 1:** Demographics and clinical characteristics of the study participants

Characteristics	Study participants (*n*=84)
Age, median years (IQR)	49 (45–56)
Age group, *n* (%)	
40–49	42 (50.0)
50–59	29 (34.5)
60-	13 (15.5)
Presumptive transmission route, *n* (%)	
Men who have sex with men	39 (46.4)
Heterosexuals	32 (38.1)
Intravenous drug users	0 (0)
Unknown	13 (15.5)
Nadir CD4 cell count, median cells/µL (IQR)	116 (45–245)
Current CD4 cell count, median cells/µL (IQR)	531 (345–762)
HIV viral load, *n* (%)	
Undetectable (<40 copies/mL)	75 (89.3)
< 400 copies/mL	8 (9.5)
≥ 400 copies/mL	1 (1.2)
Time since HIV diagnosis, median months (IQR)	92 (43–135)
Duration of antiretroviral therapy, median months (IQR)	71 (36–120)
Positive for hepatitis B antigen, *n* (%)	7 (8.3)
Positive for hepatitis C antibodies, *n* (%)	3 (3.6)
Diabetics	15 (17.9)
Hyperlipidemia	29 (34.5)
Body mass index, median kg/m^2^ (IQR)	22.5 (20.7–24.3)
Smoking, *n* (%)	
None	50 (59.6)
< 1 pack/day	17 (20.2)
≥ 1 pack/day	17 (20.2)
Alcohol consumption currently, *n* (%)	
< 60 kcal/day	64 (76.2)
≥ 60 kcal/day	20 (23.8)
Exercise, *n* (%)	
None	59 (70.2)
Running more than one hour in a week	25 (29.8)
Serum 25(OH) vitamin D level, *n* (%)	
Normal (>30 ng/mL)	7 (8.3)
Insufficiency (20–30 ng/mL)	22 (26.2)
Deficiency (<20 ng/mL)	55 (65.5)
Serum carboxyl-terminal collagen crosslinks level, *n* (%)	
Normal (<0.3 ng/mL)	17 (20.2)
Elevated (≥0.3 ng/mL)	67 (79.8)
Parathyroid hormone, median pg/mL (IQR)	49.9 (42.8–68.3)
Bone-specific alkaline phosphatase, IU/L (IQR)	33.1 (27–42.1)
Total testosterone, ng/mL (IQR)	4.7 (3.7–6.5)
Free testosterone, pg/mL (IQR)	10.4 (7.4–14.4)

IQR, interquartile range; HIV, human immunodeficiency virus.

**Figure 1:**
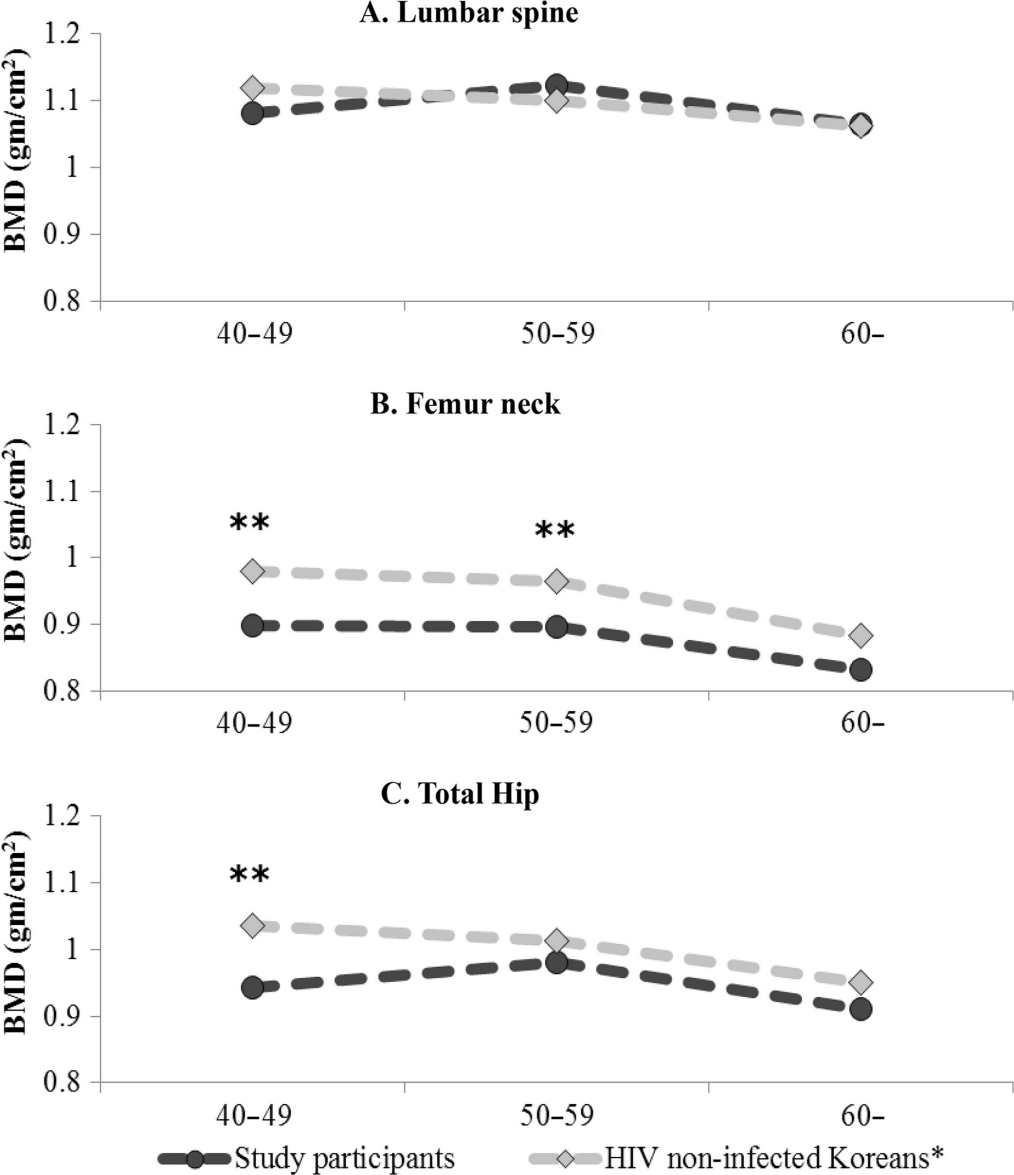
Comparison of the bone mineral densities of the study participants with those of non-HIV-positive Koreans. *Data from the Ansung cohort study [6]. **p<0.05.

The overall prevalence of low bone mass was 16.7% in the 40–49 years age group and 54.8% in the >50 years age group (Table 2). Compared with HIV-negative Koreans, the subjects in our cohort with HIV infection had significantly lower bone mass at femur neck and total hip in the 40–49 years age group (Figure 1B and 1C). In the 50–59 year age group, subjects with HIV infection had significantly lower bone mass at the femur neck than HIV-negative Koreans. There was no significant difference in the BMD at lumbar spine between HIV-negative Koreans and our subjects.

**Table 2:** Prevalence of low bone mass in the study participants

	Normal	Low BMD for chronological age^a^	
40–49 age group			
Lumbar spine	35 (83.3)	7 (16.7)	
Femoral neck	42 (100)	0 (0)	
Total hip	42 (100)	0 (0)	

	**Normal**	**Osteopenia** b	**Osteoporosis** b

50–59 age group			
Lumbar spine	22 (75.9)	5 (17.2)	2 (6.9)
Femoral neck	20 (69.0)	9 (31.0)	0 (0)
Total hip	27 (93.1)	2 (6.9)	0 (0)
60+ age group			
Lumbar spine	5 (38.5)	7 (53.8)	1 (7.7)
Femoral neck	7 (53.8)	6 (46.2)	0 (0)
Total hip	11 (84.6)	2 (15.4)	0 (0)

a Diagnosed using the International Society for Clinical Densitometry (ISCD) Z-score criteria (low BMD for chronological age, Z-score ≤ − 2.0).

b Diagnosed using the World Health Organization (WHO) T-score criteria (Osteopenia, −2.5 < T-score < − 1.0; Osteoporosis, T-score ≤ − 2.5). BMD, bone mineral density.

The association between BMD and other factors was investigated. There was no significant association between BMD and HIV exposure category (between MSM and heterosexual men), CART category (between protease inhibitor-based and non-nucleoside reverse transcriptase inhibitor-based), smoking, alcohol intake or exercise status. BMD was positively associated with BMI at all three sites and these associations were independent of other factors (Table 3). BMD at the femoral neck and total hip was negatively associated with serum CTX and these associations were independent of other factors. Bone specific ALP was negatively associated with lumbar spine BMD in multivariate analysis. The logistic regression analysis revealed that independent risk factors for low BMD were low BMI, high serum CTX, and high bone-specific ALP (Table 4).

**Table 3:** Summary of linear regression analysis of factors associated with bone mineral density

	Lumbar spine				Femoral neck				Total hip			
			
	Univariate		Multivariate		Univariate		Multivariate		Univariate		Multivariate	
						
Variables	B	*p*	B	*p*	B	*p*	B	*p*	B	*p*	B	*p*
Age	−0.001	0.752	**–**	**–**	−0.002	0.206	**–**	**–**	−0.015	0.988	**–**	**–**
BMI	0.031	<0.001	0.028	<0.001	0.019	<0.001	0.016	0.002	0.024	<0.001	0.021	<0.001
Smoking	−0.003	0.910	**–**	**–**	0.001	0.963	**–**	**–**	0.003	0.859	**–**	**–**
Alcohol (≥60 kcal/day)	−0.078	0.062	−0.050	0.178	−0.029	0.369	**–**	**–**	−0.031	0.369	**–**	**–**
Exercise^a^	−0.050	0.198	**–**	**–**	−0.023	0.436	**–**	**–**	−0.039	0.218	**–**	**–**
PI-based regimen ever	0.027	0.535	**–**	**–**	0.015	0.645	**–**	**–**	0.037	0.301	**–**	**–**
ART duration	0.000	0.393	**–**	**–**	0.000	0.243	**–**	**–**	0.000	0.359	**–**	**–**
Nadir CD4	−0.001	0.630	**–**	**–**	−0.002	0.854	**–**	**–**	−0.001	0.680	**–**	**–**
Current CD4	0.001	0.681	**–**	**–**	−0.001	0.787	**–**	**–**	0.001	0.740	**–**	**–**
25(OH) Vitamin D3	0.001	0.733	**–**	**–**	−0.001	0.787	**–**	**–**	0.001	0.751	**–**	**–**
PTH	0.001	0.111	**–**	**–**	0.001	0.976	**–**	**–**	0.000	0.631	**–**	**–**
CTX	−0.261	0.002	−0.169	0.032	−0.206	0.002	−0.164	0.009	−0.233	0.001	−0.168	0.008
Bone-specific ALP	−0.002	0.112	**–**	**–**	−0.001	0.210	**–**	**–**	−0.001	0.161	−0.001	0.086
Testosterone	0.000	0.971	**–**	**–**	−0.008	0.253	**–**	**–**	−0.006	0.420	**–**	**–**
Free testosterone	−0.002	0.676	**–**	**–**	−0.002	0.393	**–**	**–**	−0.002	0.566	**–**	**–**
Urine Ca/Cr	−0.106	0.710	**–**	**–**	−0.149	0.492	**–**	**–**	−0.158	0.495	**–**	**–**

a Running more than one hour a week.

BMI, body mass index; PI, protease inhibitor; PTH, parathyroid hormone; CTX, carboxy-terminal collagen crosslinks; ALP, alkaline phosphatase; Ca, calcium; Cr, creatinine.

**Table 4:** Summary of logistic regression analysis of factors associated with low bone mineral density (any T score≤ − 1)

	Univariate			Multivariate		
		
Variables	OR^a^	95% CI	*p*	aOR^a^	95% CI	*p*
Age	1.330	0.847–2.089	0.215	**–**	**–**	**–**
BMI	0.479	0.285–0.805	0.005	0.466	0.256–0.849	0.013
Smoking	0.961	0.562–1.642	0.883	**–**	**–**	**–**
Alcohol (≥60 kcal/day)	3.000	0.974–9.236	0.056	**–**	**–**	**–**
Exercise^b^	1.606	0.613–4.208	0.335	**–**	**–**	**–**
PI-based regimen ever	0.399	0.107–1.483	0.170	**–**	**–**	**–**
ART duration	1.004	0.651–1.548	0.987	**–**	**–**	**–**
Nadir CD4	1.293	0.829–2.018	0.258	**–**	**–**	**–**
Current CD4	1.009	0.654–1.556	0.968	**–**	**–**	**–**
25(OH) Vitamin D3	0.932	0.604–1.437	0.749	**–**	**–**	**–**
PTH	1.050	0.680–1.623	0.825	**–**	**–**	**–**
CTX	3.125	1.572–6.211	0.001	2.672	1.325–5.388	0.006
Bone-specific ALP	1.996	1.016–3.923	0.045	2.325	1.021–5.291	0.044
Testosterone	1.268	0.810–1.986	0.300	**–**	**–**	**–**
Free testosterone	1.200	0.774–1.861	0.414	**–**	**–**	**–**
Urine Ca/Cr	1.238	0.788–1.946	0.354	**–**	**–**	**–**

a Odds ratios are shown as per one standard deviation increment.

b Running more than one hour in a week.

OR, Odds ratio; CI, confidence interval; BMI, body mass index; PI, protease inhibitor; PTH, parathyroid hormone; CTX, carboxy-terminal collagen crosslinks; ALP, alkaline phosphatase; Ca, calcium; Cr, creatnine.

## Discussion

In the present study, BMDs at the lumbar spine, femur neck and total hip were investigated among Korean HIV-positive male patients undergoing CART, and clinical factors and serum markers were measured. Low bone mass was prevalent among Korean HIV-positive male patients undergoing antiretroviral treatment. Several factors were associated with low bone mass.

A high prevalence of low bone mass has been reported in HIV-positive individuals in many cross-sectional studies [7, 8]. The prevalence of osteoporosis was more than three times greater among HIV-positive patients than among HIV-negative control subjects in a meta-analysis [4]. A decrease of BMD ranging from −1.5 to −5.8%, depending on the antiretroviral regimen, was observed within the first year of antiretroviral therapy [9]. The overall prevalence of fractures was significantly higher in HIV-positive patients, than in HIV-negative patients (2.87 vs. 1.77 fractures per 100 persons) [10]. Accordingly, in the present study, the patients with HIV infections had significantly lower BMD than age-matched HIV-negative Koreans in the general population. In the present study, the overall prevalence of osteoporosis and osteopenia were 9.5 and 46.4%, respectively. The reported prevalence of osteoporosis in HIV-positive patients varies widely according to age group and gender (3–33%) [3]. Several researchers have reported the prevalence of osteoporosis in HIV-positive men in Western countries (France: 16%, mean age 40 years; Australia: 3%, mean age 43 years) [7, 11]. A recent study reported the prevalence of osteoporosis in HIV-positive men in Taiwan (3.1%; median age 36.5 years) [12]. However, as discussed by its authors, the Taiwanese study was limited by several factors (majority of the subjects were middle aged, no bone turnover markers were measured and, most importantly, only lumbar spine BMD was measured, not femur neck or total hip). In addition to these limitations, due to the study design (which excluded potential osteoporosis patients), the authors state that the prevalence of osteoporosis was underestimated. In the present study, the prevalence of osteoporosis was determined using all three BMD sites. And the BMDs of HIV-positive men were compared with those of age-matched same-race controls. The association between BMD and bone-metabolism-related factors including bone turnover markers was also investigated.

The causes of low bone mass in HIV appear to be multifactorial. First, there are complex interactions between HIV infection and traditional osteoporosis risk factors [13]. Chronic HIV infection leads to poor nutrition and low weight. Indeed, the BMI of the HIV-negative control group was 23.8±3.1 and the BMI of the HIV-positive study group was 22.6±2.5. This difference in BMI could explain some portion of the difference in BMD. Low vitamin D levels [14] and hypogonadism [15] are associated with HIV infection. The prevalence of diabetes was high among the study subjects. The high prevalence of diabetes could have a substantial effect on bone metabolism. However, the BMD was not significantly different between diabetic and non-diabetic patients and similar trends were observed when diabetic subjects were excluded. High rates of alcohol and tobacco use are reported among HIV-positive patients. Second, uncontrolled HIV viremia per se may decrease bone mass [4, 16]: systemic inflammation caused by HIV infection can affect bone remodelling. There have been several studies of the association between HIV infection and bone remodelling. HIV proteins have been reported to augment bone resorption through increased osteoclast activity [17], and HIV proteins attenuate bone formation by stimulating osteoblasts apoptosis [18]. Third, CART-related factors may also link low bone mass and HIV infection. Continuous CART decreased BMD [19] and intermittent CART resulted in less loss of BMD [20]. These complex mechanisms could have resulted in the low BMD observed in the present study.

We found that low BMI was associated with low BMD at all three sites. Similar findings were obtained in previous studies [11, 12], demonstrating the important association between body weight and BMD. Markers of bone metabolism (CTX and bone-specific ALP) were negatively associated with BMD in the present study. This is in accordance with previous studies of HIV-positive patients [21, 22], suggesting that high bone turnover is associated with the reduction in BMD. The initiation of CART led to an increase in markers of bone turnover [22] and intermittent application of CART reduced the decline in markers of bone turnover and in BMD [20], suggesting that CART decreases BMD by increasing bone turnover. These findings indicate that measurements of bone turnover markers in HIV-positive patients could be used in the clinic to assess bone metabolism status in HIV.

In the present study, we observed significantly lower femur neck BMD and total hip BMD in HIV-positive patients than in HIV-negative patients in the general Korean population. However, no significant difference was observed in lumbar spine BMD. A previous study also reported that the difference in lumbar spine BMD was borderline whereas the difference in hip BMD was significant [7]. Others have reported that both lumbar spine BMD and femur neck BMD are significantly lower in HIV-positive patients [8].

A recent study showed that intermittent CART has less effect on BMD than continuous antiretroviral therapy [20], which suggests a deleterious effect of CART on bone. Also, longer duration of CART was associated with reduced BMD [12]. However, in the present analysis, the duration of CART was not associated with BMD.

In one recent study, the prevalence of low BMD was similar among HIV-positive MSM and HIV-negative MSM [23], suggesting that the low BMD found in the former may precede HIV acquisition. Compared with heterosexual men, MSM are likely to have the conventional risk factors for osteoporosis (such as low body weight, alcohol use, smoking) [24]. Based on these findings, it has been suggested that the low BMD found among HIV-positive MSM may be the result of MSM-related factors, and may not be fully attributable to HIV infection alone or the use of ART [23]. However, in the present study, there was no significant difference in BMD between MSM and heterosexual men, suggesting that the low BMD is more likely to be the result of HIV infection and/or the use of CART than MSM-related life style factors.

This study has several limitations. First, because of its cross-sectional design, causal relationships between HIV infection and reduced BMD could not be determined. Second, its statistical power was low due to the small sample size. Third, half of the study subjects were under the age of 50 years. Inclusion of these young participants could have attenuated the statistical significance regarding the effect of ageing on bone loss. However, to the best of our knowledge, this is the first study in the English language to report the prevalence of osteoporosis based on all three BMD sites as well as factors related to bone metabolism, including bone turnover markers, in HIV-positive Asian men.

## Conclusions

In this first survey for HIV-associated osteopenia in Asians undergoing antiretroviral therapy, the prevalence of low bone mass was significantly higher compared to non HIV-infected individuals. Low bone mass was associated with increased bone resorption.
